# Concurrent development of testicular seminoma and choriocarcinoma of the superior mediastinum, presented as cervical mass: a case report and implications about pathogenesis of germ-cell tumours

**DOI:** 10.1186/1472-6890-6-8

**Published:** 2006-11-20

**Authors:** Giannis Mountzios, George Pavlakis, Evangelos Terpos, George Sakorafas, Kyriakos Revelos, Aristotelis Bamias, Nikolaos Nikolaou, Pantelis Papasavas, Jean-Charles Soria, Meletios-Athanasios Dimopoulos

**Affiliations:** 1 Medical Oncology Dpt, "Alexandra" University Hospital School of Medicine, Athens, Greece; 2 Medical Oncology Dpt., 251 General Air force Hospital, Athens, Greece; 3 Dpt. Of Haematology, 251 General Air force Hospital, Athens, Greece; 4 1st Dpt. of Surgery, 251 General Air force Hospital, Athens, Greece; 5 Dpt of Pathology, 251 General Air force Hospital, Athens, Greece; 6 Dpt. of Biomedical Research 251 General Air force Hospital Athens, Greece; 7 Dpt. Of Medicine, Institut Gustave Roussy, Villejuif, France

## Abstract

**Background:**

Synchronous presentation of more than one germ cell tumours of different histology in the same patient is considered to be very rare. In these cases of multiple germ cell tumours, strong theoretical and clinical data suggest an underlying common pathogenetic mechanism concerning genetic instability or abnormalities during the pluripotent embryonic differentiation and maturation of the germ cell.

**Case presentation:**

A 25 year-old young man presented with an enlarging, slightly painful left cervical mass. Despite the initial disorientation of the diagnosis to a possible thyroid disorder, the patient underwent complete surgical resection of the mass revealing mediastinal choriocarcinoma. Subsequent ultrasound of the scrotum indicated the presence of a small lobular node in the upper pole of the left testicle and the patient underwent radical left inguinal orchiectomy disclosing a typical seminoma. Based on these results, the patient received 4 cycles of Bleomycin, Etoposide and Platinum chemotherapy experiencing only mild toxicity and resulting in complete ongoing clinical and biochemical remission.

**Conclusion:**

The pathogenesis of concurrent germ cell tumours in the same patient remains an area of controversy. Although the genetic instability of the pluripotent germ cell offers an adequate explanation, the possibility of metastasis from the primary, less differentiated tumour to a distant location as a more mature subtype cannot be excluded. Possible development of a metastatic site of different histology and thus biological behaviour (e.g choriocarcinoma) should be anticipated. Furthermore, urologists, pathologists and medical oncologists should be meticulous in the original pathological diagnosis in these patients, since there is a significant frequency of germ cell tumours with mixed or overlapping histological elements with diverse potential of evolution and differentiation.

## Background

Synchronous presentation of more than one germ cell tumors (GCTs) in the same patient is considered to be quite rare. It usually involves almost identical histological subtypes, the possibility of metastasis from the one site to another being the most probable explanation [1]. Here we describe a case of concurrent presentation of left testicular seminoma and choriocarcinoma of the upper mediastinum presenting as an enlarging, painless, left cervical mass.

## Case presentation

**Figure 1:**
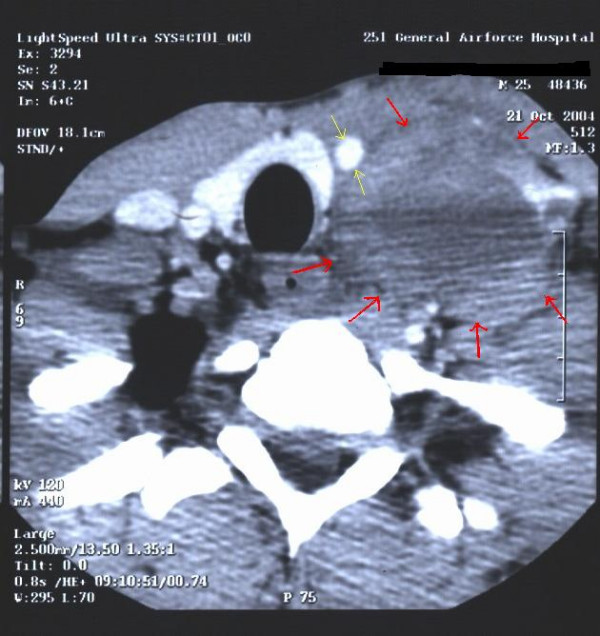
CT scan of the cervix revealing a large mass (red arrows), 14 cm in greatest diameter extending from the left carotide triangle to the anterior-posterior mediastinum, in proximity with the great vessels of the heart, dislocating the left common carotid artery (yellow arrows) and the left vagus nerve without infiltrating them.

**Figure 2:**
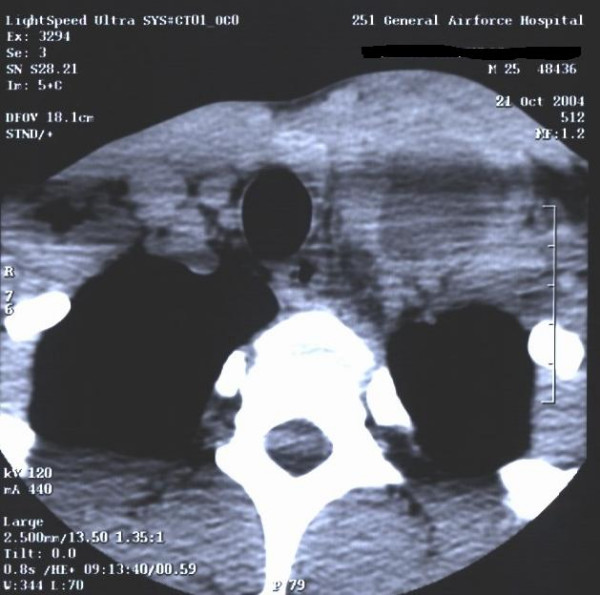
CT scan of the upper mediastinum (complementary to figure 1).

**Figure 3:**
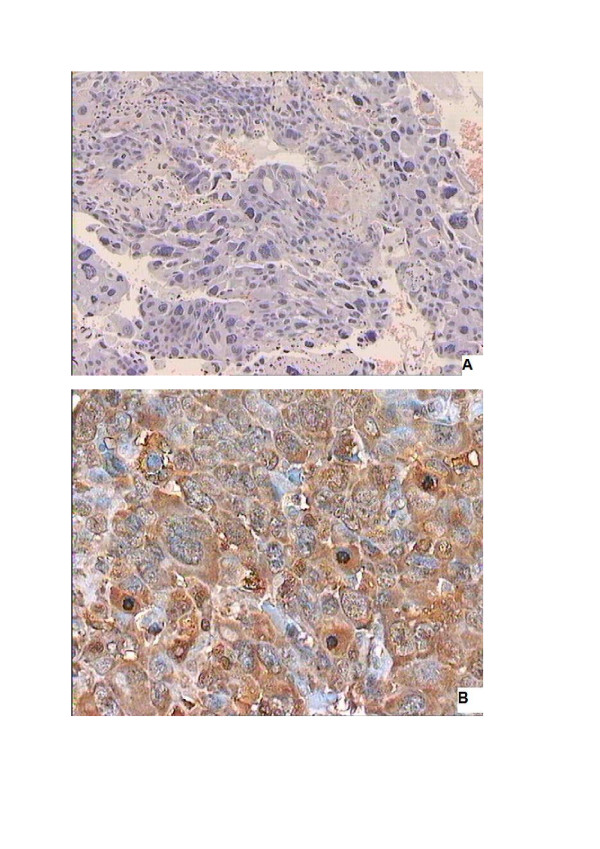
A. Mediastinal choriocarcinoma: Diffuse sheets of cytotrophblastic and syncytiotrophoblastic cells. Hemorrhagic and necrotic locations are also seen. (medium power magnification) B. Tumor cells with diffuse positive cytoplasmic immunostaining for β-hCG.(High power magnification).

**Figure 4:**
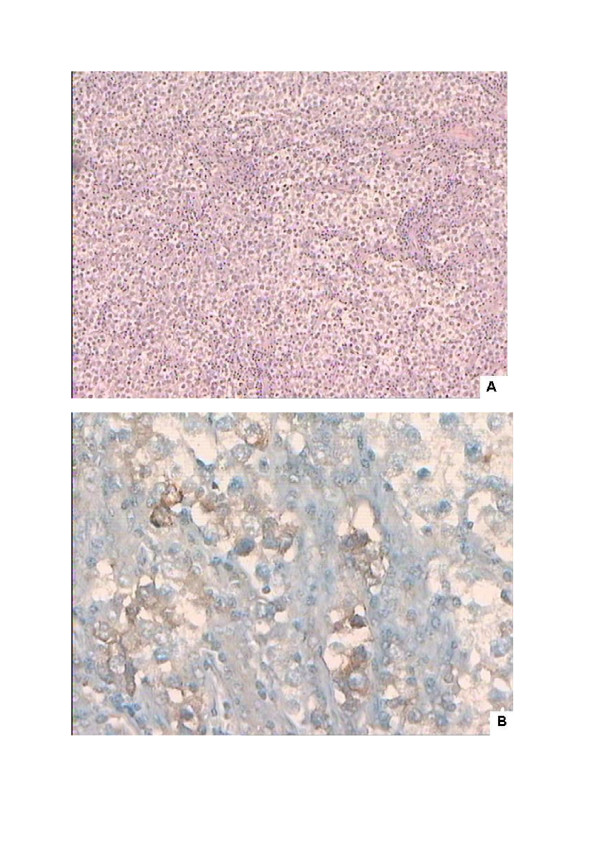
A. Testicular seminoma: Uniform tumor cells with abundant clear cytoplasm and centrally located nucleus. Tumor nests are outlined by fibrous bands infiltrated by lymphocytes. (medium power magnification). 4B: Rare cells stain positive for β-hCG (high power magnification).

**Figure 5:**
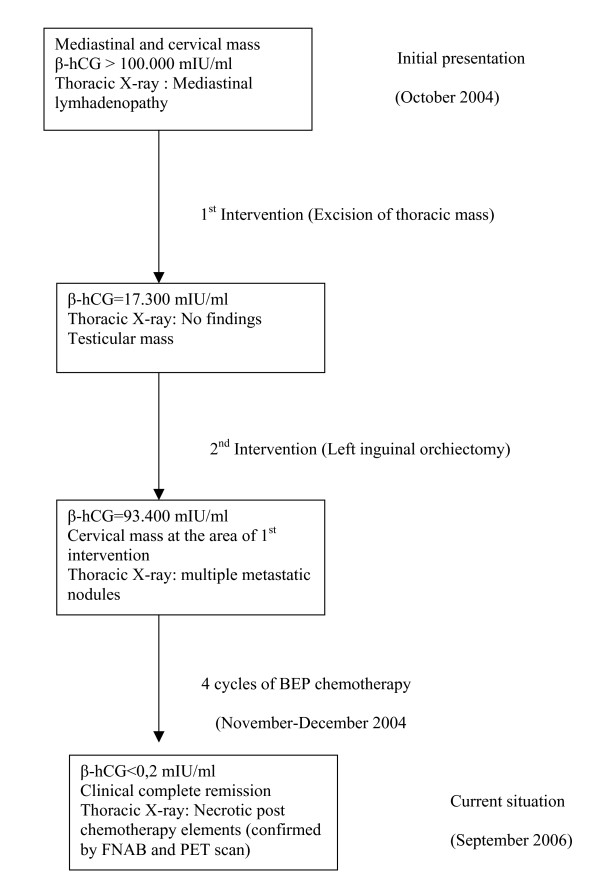
Schematic presentation of the diagnosis and treatment course, including β-CG title and X-ray findings. FNAB: fine-needle aspiration biopsy.

A 25-year-old male was presented in October 2004 with a monthly history of a gradually enlarging, painless, left cervical mass. He had no history of prior thyroid disorder or other disease. Clinically the mass was hard in palpation, firmly attached to the surrounding tissues and was located at the anatomic area of the carotide triangle. Thorough physical examination revealed only a marginal hepatomegaly and splenomegaly. Routine blood tests, biochemical markers and thyroid function tests – including triiodothyronine (T_3_), thyroid stimulating hormone (TSH), thyreoglobulin and anti-thyroid antibodies- were within normal limits, with the exception of a moderately elevated erythrocyte sedimentation rate (ESR = 44). Subsequent cervical and thoracic CT scan revealed a multilobular mass (4,3 × 4,7 × 7 cm) probably arising from the left thyroid lobe, with infiltrating features and heterogeneous density with regions of central necrosis and hemorrhage. The mass submerged into the anterior-posterior mediastinum, in proximity with the great vessels of the heart, dislocating the left common carotid artery and the left vagus nerve without infiltrating them (Fig. 1 and 2). Significant mediastinal lymphadenopathy was also noted, whereas ultrasound of the abdomen excluded liver involvement. Differential diagnosis included thyroid carcinoma, lymphoma, thymoma, malignant congenital branchiac cyst or cystic hygroma and germ cell tumours of the upper mediastinum. Fine-needle aspiration biopsy of the cervical mass was performed and the cytological findings were consistent with papillary thyroid carcinoma with anaplastic features. Based on these findings, the patient was referred for surgical removal of the lesion. At surgery, a large mass, measuring 9,5 × 6,3 × 4,5 cm was found behind the left srenocleidomastoid muscle, located lateral to the left carotid artery/jugular vein and was not firmly adhered to the left thyroid lobe. The mass was easily separated from the surrounded tissues and was removed. Based on the results of preoperative fine needle aspiration biopsy, a total thyroidectomy was performed at the same time. Pathology examination of the mass revealed extensive infiltration by large or giant malignant cells with morphological features consistent with syncytiotrophoblasts within necrotic and hemorrhagic elements (Fig. 3A). Positive immunohistochemical staining with β-subunit of human chorionic gonadotrophin (β-hCG) suggested the diagnosis of choriocarcinoma of the upper mediastinum (Fig. 3B). The pre-operation serum concentration of β-hCG was >100.000 mIU/ml, whereas the immediate post-operation levels declined to 17.300 mIU/ml. Alpha- fetoprotein (a-FP) and carcinoembryonic antigen (CEA) levels were within normal limits, while lactate dehydrogenase (LDH) level was two-fold higher than the normal upper limit. Based on these data, scrotal ultrasound examination was performed, disclosing a small multilobular mass measuring 2,1 cm in greatest diameter-not evident at previously performed physical examination- located on the upper pole of the left testicle with echomorphological and hemodynamic characteristics consistent with seminomatous tumour. Subsequently, the patient underwent left radical inguinal orchiectomy with high ligation of the left spermatic cord and implantation of synthetic testicular prothesis. Histological diagnosis of the testicular tumour revealed almost typical seminoma (1,8 × 1,2 × 1 cm) consisting of large clear-cytoplasm cells with hypodense nucleus and a few atypical mitosis, without any signs of infiltration of *rete testis *or the spermatic cord. Complete inhibition of spermatogenesis and hyperplastic reaction of Leydig cells were also observed (Fig. 4A). Although immunohistochemical staining for β-hCG was positive in a few cells, their morphological characteristics did not meet the diagnostic criteria for syncytiotrophoblasts (Fig. 4B). On postoperative day six (6), the patient developed slightly painful cervical mass at the anatomic site of the first surgical intervention and β-hCG levels started rising up again (β-hCG = 29.850 mIU/ml) (Fig. 5). Complete pre-therapeutic staging was immediately performed, including negative CT scan of the brain and negative bone scan, whereas CT scan of the thorax and the abdomen disclosed multiple round metastatic nodules of various size (0,1 – 2 cm) in both lungs and marginally enlarged iliac and para-aortic lymph nodes without liver or other parenchymal organ involvement. In November 2004, one month after his initial admission to the hospital, the patient received 1^st ^line chemotherapy for high-risk germ cell tumour with the BEP regimen (Bleomycin 30 mg: d_1_-d_8_-d_15_, Etoposide 100 mg/m^2^: d_1_-d_5 _and Cisplatin 20 mg/m^2^: d_1_-d_5 _in 21-day cycles). Pre-chemotherapy levels of β-hCG were 93.400 mIU/ml. The patient completed 4 cycles of therapy without experiencing remarkable toxicity (Neutropenia grade I-II according to the NCI-CTC criteria) and is currently (October 2006) asymptomatic, with ongoing complete clinical and biochemical remission according to the RECIST criteria (No evidence of tumour mass, regression of all enlarged lymph nodes, necrotic post-chemotherapy elements in the remaining lung nodules confirmed by CT-guided fine-needle aspiration biopsy and PET scan and consecutively normal levels of β-hCG). A schematic presentation of the whole diagnosis and treatment course including β-hCG titer and chest Xray findings is illustrated in Fig. 5

## Discussion

GCTs are generally malignant and represent 93% of all testicular neoplasms [1]. At least half of them are of seminomatous cell origin, being either "typical" seminomas or containing mixed elements of non-seminomatous origin (embryonal or choriocarcinoma elements such as syncytiotrophoblasts) which do not usually affect the good prognosis of seminomas [2]. On the other hand, non-seminomatous GCTs, represent a more heterogenous group comprising four main subtypes (embryonal carcinoma, teratoma, choriocarcinoma and yolk sac tumor) with significant overlapping and increased frequency of mixed histological pictures [3].

It is thought today that these common histological elements between seminomatous and non-seminomatous GCTs and also between the different subtypes of non-seminomatous GCTs reflect their common embryonic origin from the same primitive, pluripotent germ cell which has the capacity to mature and differentiate to neoplastic endodermal or ectodermal components, imitating thus the procedures of normal embryonic development [1]. Consequently, each type of germinal cancer is considered to be the "counterpart" of each stage of normal embryo development: Seminoma is the neoplastic counterpart of the spermatocyte and represents the more undifferentiated type. The next stage is that of the fertilized ovum and the formation of the blastocele which gives rise to both the embryo and the placenta: the neoplastic counterpart is the embryonal cell carcinoma, which produces high levels of a-FP concentration. At a more mature stage of embryonic development, malignant transformation of the developing embryo will lead to teratomas (a-FP and β-hCG production), whereas neoplastic transformation of the embryonic yolk sac cells leads to the homonymous tumours, overproducing a-FP. Finally, syncytiotrophoblastic and cytotrophoblastic components of the placenta will give rise to pure choriocarcinomas, that overproduce β-hCG. Although choriocarcinomas represent a more differentiated malignant counterpart, they are characterised by an aggressive biological behaviour with tendency for haematogenous metastasis, probably reflecting the capacity of its normal counterpart (the placenta) to invade blood vessels [4]. The last observation suggests that malignant transformation of a more mature element during embryonic development does not necessarily predict either a benign biological behaviour of the tumour or a more favourable clinical outcome.

Based on the above mentioned data, the hypothesis that the concomitant development of two different histological types of germ cell tumour in the same patient is random, does not seem probable. Interestingly, synchronous or metachronous development of multiple germ cell tumours in the same patient insinuates an underlying common pathogenetic mechanism concerning genetic instability or abnormalities during the pluripotent embryonic germ cell differentiation and maturation. A number of genetic abnormalities, the most common being isochromosome 12, have been described in patients with germ cell tumours and have also been implemented in the diagnostic procedure of undifferentiated mediastinal tumours of uncertain histological origin [5]. It seems that germ cells of the testicles (or the ovaries), as well as the primitive embryonic germ cells that have remained in extragonadal locations in the midline of the body (mediastinum, retroperitoneum), can undergo malignant transformation and mimic procedures of normal embryonic development by differentiating towards various embryonic or extra-embryonic elements.

Recently, it has been suggested that the synchronous or metachronous presentation of different types of GCTs in the same patient does not indicate two different primary tumours with common pathogenetic origin, but the more differentiated site might represent a possible metastatic location of the primary undifferentiated tumour, which has metastasised as a more mature type [4]. Interestingly enough, this histological "conversion" into a more differentiated type at the metastatic location has been observed in a few series of patients with GCTs [6]. We know today that metastatic sites are not always histologically identical with the primary location: histological conversion can occur, either as a normal maturation procedure mimicking embryonic development or as a result of therapeutic intervention. In the last case, chemotherapy can destroy the rapidly growing, chemosensitive embryonal components of a mixed GCT, sparing the more resistant teratomatous elements and resulting thus in a completely different histology compared to that of the primary tumour location [4].

The above mentioned hypothesis implies that GCTs may metastasise only as a more mature histological type in the procedure of embryogenesis and never in the opposite direction, which has been confirmed by numerous clinical observations: Metastases from embryonic carcinomas may be found to consist of both teratoma and choriocarcinoma elements, whereas choriocarcinomas metastasise only as choriocarcinomas, since they represent the more differentiated histological type of embryonic development [6]. Seminomas possess the theoretical capacity of metastasising as various histological components since they represent the malignant counterpart of the more "primitive" cell, the spermatocyte. However, some authors suggest that "typical" seminomas always metastasise keeping their original histological features: those who do not are believed to represent GCTs of mixed histology, misdiagnosed as seminomas at the original histological examination. Supporters of this theory believe that histological "conversion" can not happen either automatically or after therapeutic intervention. Ayala and Ro suggest that all GCTs, including seminomas, consist of all embryonic elements, not always detectable at initial pathology examination and have thus the potential of giving rise to histologically different metastases consisting of one of the primary tumour elements, depending on their different metastatic potential and the therapeutic intervention including chemotherapy and radiotherapy [1].

Our patient developed almost concurrently testicular seminoma and pure choriocarcinoma of the mediastinum. Histological "conversion" of the original tumour site is theoretically possible since choriocarcinoma represents a more mature stage of embryonic development compared to seminoma. On the other hand, one could argue that the primary tumour was not a typical seminoma and that chorionic elements where present at the original histological examination including few giant cells with positive immunohistochemistry for β-hCG. Nevertheless, trophobalstic elements, whose presence is considered to be mandatory for the diagnosis of choriocarcinoma, were totally absent in the original pathology specimen.

The above mentioned potential of GCTs to metastasise as a more differentiated subtype is clinically meaningful, since the clinician should anticipate possible development of a metastatic site of a more aggressive biological behaviour (e.g. choriocarcinoma) compared to that of the original location. Thus, medical oncologists, urologists and pathologists should be very suspicious of the original pathological diagnosis in these patients, since there is a significant frequency of GCTs with mixed or overlapping histological elements with diverse potential of evolution and differentiation. Moreover, given the heterogeneity of these tumours, one could emphasize the necessity of obtaining multiple samples from different tumour areas in every case of GCT so as to minimize the risk of missing important particular components of the tumour who could potentially affect the diagnosis, prognosis or even the therapeutic intervention.

The presenting symptom of our patient was an enlarging, painless cervical mass, originally thought to be a thyroid node and is considered to be an extremely rare presentation. With the exception of choriocarcinoma, which gives early haematogenous metastases, testicular tumours usually become apparent in their primary location with painless or slightly painful enlargement of the testicle noticed accidentally by the patient himself. Discolo and Dispaquale reported a case of testicular seminoma with cervical lymphadenopathy as the presenting symptom [7]. Costal bones, brain, spleen, paracolic gutter, urethra, inferior vena cava, pancreatic and subcutaneous tissue have been reported as rare metastatic locations of primary testicular cancer but not as the presenting manifestation [8-14] (table 1). In our patient the painless cervical enlargement was due to the growing mediastinal choriocarcinoma and is, to our knowledge, the first case of concurrent testicular seminoma and mediastinal choriocarcinoma ever reported. The unusual location, along with the results of fine-needle aspiration biopsy disorientated initially the diagnosis towards malignancy of the thyroid gland. Thyroid carcinoma with anaplastic features is considered to be extremely rare in young males and the clinician should be very suspicious in every mediastinal mass of uncertain histology in young adults. Detailed examination of the testicles including scrotal ultrasound with Doppler angiography should be performed in every diagnosed or highly suspected extra-gonadal germ cell tumour, as the latter could represent a possible metastatic site. Complete staging along with solid histological confirmation in both tumour locations are mandatory before any therapeutic intervention is initiated.

## Conclusion

Germ cell tumours represent a heterogeneous group of malignant cell lines with a variety of frequently overlapping histological pictures or with mixed components suggesting a common "precursor" embryonic cell dysfunction. Histological conversion to a more mature subtype is theoretically possible in a metastatic location with or without therapeutic intervention, as well as synchronous or metachronous development of two different primary germ cell tumours as a result of a common pathogenetic mechanism concerning genetic instability or abnormalities during the pluripotent embryonic germ cell differentiation and maturation.

## Competing interests

The authors have not any potential conflicts of interest to disclose. G.M. is a European Society of Medical Oncology (ESMO) fellowship recipient for the year 2005–2006.

## Authors' contributions

GM conceived of the study, obtained and analysed the data and wrote the manuscript. GP participated in the design of the study and the analysis of the data. ET participated in the design of the study and the writing of the manuscript.

## Pre-publication history

The pre-publication history for this paper can be accessed here:



## Figures and Tables

**Table 1:** Summary of the published cases of synchronous or metachronous development of second GCT in patients with primary testicular cancer.

TYPE OF PRESENTATION	LOCALISATION OF SECOND TUMOUR	HISTOLOGY OF SECOND TUMOUR	REFERENCE
Metachronous	Costal bones	SGCT	[8]
Metachronous	Brain and Spleen	NSGCT	[9]
Metachronous	Paracolic	SGCT	[10]
Synchronous	IVC and GI	SGCT	[11]
Metachronous	Pancreas	NSGCT	[12]
Synchronous	Mediastinal	NSGCT	[13]
Metachronous	Subcutaneous	NSGCT	[14]

SGCT: seminomatous germ-cell tumours, NSGCT: non-seminomatous germ-cell tumours, IVC: inferior vena cava, GI: Gastro-intestinal tract.
